# A survey of BWT variants for string collections

**DOI:** 10.1093/bioinformatics/btae333

**Published:** 2024-05-24

**Authors:** Davide Cenzato, Zsuzsanna Lipták

**Affiliations:** Department of Environmental Sciences, Informatics and Statistics, Ca’ Foscari University, Venice, 30123, Italy; Department of Computer Science, University of Verona, Verona, 37134, Italy

## Abstract

**Motivation:**

In recent years, the focus of bioinformatics research has moved from individual sequences to collections of sequences. Given the fundamental role of the Burrows–Wheeler transform (BWT) in string processing, a number of dedicated tools have been developed for computing the BWT of string collections. While the focus has been on improving efficiency, both in space and time, the exact definition of the BWT used has not been at the center of attention. As we show in this paper, the different tools in use often compute non-equivalent BWT variants: the resulting transforms can differ from each other significantly, including the number *r* of runs, a central parameter of the BWT. Moreover, with many tools, the transform depends on the input order of the collection. In other words, on the same dataset, the same tool may output different transforms if the dataset is given in a different order.

**Results:**

We studied 18 dedicated tools for computing the BWT of string collections and were able to identify 6 different BWT variants computed by these tools. We review the differences between these BWT variants, both from a theoretical and from a practical point of view, comparing them on eight real-life biological datasets with different characteristics. We find that the differences can be extensive, depending on the datasets, and are largest on collections of many similar short sequences. The parameter *r*, the number of runs of the BWT, also shows notable variation between the different BWT variants; on our datasets, it varied by a multiplicative factor of up to 4.2.

**Availability and implementation:**

Source code and scripts to replicate the results and download the data used in the article are available at https://github.com/davidecenzato/BWT-variants-for-string-collections.

## 1 Introduction

The Burrows–Wheeler transform (BWT) (Burrows and Wheeler 1994) is a fundamental string transformation which is at the heart of many modern compressed data structures for text processing, in particular in bioinformatics (Langmead *et al.* 2009, Li and Durbin 2010, Langmead and Salzberg 2012). With the increasing availability of low-cost high-throughput sequencing technologies, the focus has moved from single strings to large string collections, such as the 1000 Genomes Project (The 1000 Genomes Project Consortium 2015), the 10 000 Genomes Project (Genome 10K Community of Scientists 2009), the 100 000 Human Genome Project (Turnbull *et al.* 2018), the 1001 Arabidopsis Project (The 1001 Genomes Consortium 2016), and the 3000 Rice Genomes Project (3K RGP) (Sun *et al.* 2017). This has led to a widespread use of compressed data structures on inputs which are collections of sequences, rather than individual sequences.

A number of tools have been developed in recent years for computing the BWT of a collection (multiset) of strings. The focus has been on efficiently processing datasets of ever increasing sizes, but little attention has been paid to the actual method used to compute the BWT. This is an issue, as the BWT was originally defined for a single string, and it is not immediately clear how to define it for a collection (multiset) of strings. In fact, there exists more than one way to compute a Burrows–Wheeler-type transform of multiple strings. Even though all these methods maintain the properties necessary for building string indexes on top of the BWT, such as reversibility and LF-property, they differ in other, important, ways.

As we will show in this paper, different tools not only apply different algorithms to compute the BWT of the input collection, but they output different transforms. Studying 18 publicly available tools, we identified six distinct BWT-variants which are computed by these tools. The tools included in this study are: BEETL (Bauer *et al.* 2013), BCR_LCP_GSA (Bauer *et al.* 2013), ropebwt2 (Li 2014), nvSetBWT (Pantaleoni 2014), msbwt (Holt and McMillan 2014), Merge-BWT (Sirén 2016), eGSA (Louza *et al.* 2017), BigBWT (Boucher *et al.* 2019), bwt-lcp-parallel (Bonizzoni *et al.* 2019), eGAP (Egidi *et al.* 2019), gsufsort (Louza *et al.* 2020), G2BWT (Díaz-Domínguez and Navarro 2021), grlBWT (Díaz-Domínguez and Navarro 2023), pfpebwt (Boucher *et al.* 2021a), cais (Boucher *et al.* 2021a), r-pfbwt (Oliva *et al.* 2023), CMS-BWT (Masillo 2023), and optimalBWT (Cenzato *et al.* 2023). In Table 1, we give the BWT variants as computed by these 18 tools on a toy example of five DNA-strings.

**Table 1.** Overview of some properties of the six BWT variants considered in this paper and the tools computing them.^a^

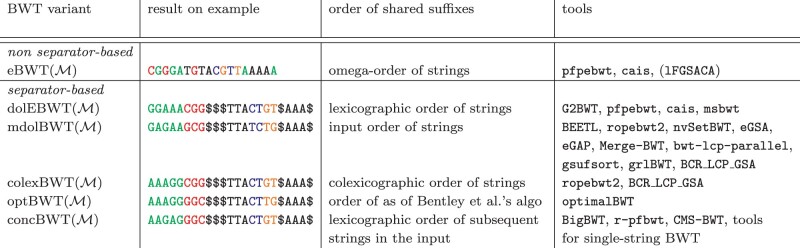			

a The colors in the example BWTs correspond to interesting intervals in separator-based variants; the same characters are highlighted in the eBWT to show their positions, see Section 3.2. (We included lFGSACA, which also computes the eBWT, in brackets, since it is a library and not a command-line tool, as opposed to all others listed.)

The size of BWT-based compressed data structures such as the RLFM-index (Mäkinen and Navarro 2005) or the *r*-index (Gagie *et al.* 2020) is typically measured in the number of runs (maximal substrings consisting of the same letter) of the BWT, commonly denoted *r*. This parameter *r* has become central as a measure of the storage space required by these data structures. Additionally, much recent research effort has concentrated on the construction of data structures which cannot only store but query, process, and mine strings in space and time proportional to *r* (Bannai *et al.* 2020, Gagie *et al.* 2020, Cobas *et al.* 2021, Oliva *et al.* 2021, Boucher *et al.* 2024). Moreover, the parameter *r* (or the related *n*/*r*, the average runlength of the BWT) is also being increasingly seen as a measure of repetitiveness of the string or strings, with several recent works theoretically exploring its suitability as such a measure, as well as its relationship to other repetitiveness measures (Giuliani *et al.* 2021, Navarro 2021, Kempa and Kociumaka 2022, Akagi *et al.* 2023). The parameter *r* is now also being used as a property of the dataset itself, e.g. (Bannai *et al.* 2020, Boucher *et al.* 2021b, Cobas *et al.* 2021).

However, the number of runs varies between the different BWT-variants, as can be seen on our toy example. This has important implications not only for the storage space required for BWT-based compressed data structures, but also for claims about the level of repetitiveness of the dataset. With competing non-equivalent methods around, this measure is not well defined. We will explore this question further (Section 4) and suggest resolving the issue by standardizing the definition.

### 1.1 Overview of methods for defining multi-string BWT

The classical way of computing text indexes of more than one string is to concatenate them, adding a different end-of-string-symbol at the end of each string, and then to compute the index for the concatenated string. This is the method traditionally used for generating classical data structures such as suffix trees resp. suffix arrays for multiple strings, and results in the so-called *generalized suffix tree* resp. *generalized suffix array*, see e.g. (Gusfield 1997, Ohlebusch 2013). Applied directly, this method would lead to an unacceptable increase in the size of the alphabet, from *σ*, often a small constant in applications, to σ+k, where *k* is the number of strings in the collection, typically in the thousands or even tens or hundreds of thousands. One way to avoid this is to use only conceptually different end-of-string-symbols, i.e. to have only one dollar-sign and apply string input order to break ties. This is the method used by most tools, including ropebwt2 (Li 2014), BCR (Bauer *et al.* 2013) (with a different algorithm which results in the same output), and many others. Another method to avoid increasing the alphabet size is to separate the input strings using the same end-of-string-symbol; in this case, a different end-of-string-symbol has to be added to the end of the concatenated string to ensure correctness, as e.g. done by BigBWT (Boucher *et al.* 2019). An equivalent solution is to concatenate the input strings without removing the end-of-line or end-of-file characters, since these act as separators; or to concatenate them without separators and use a bitvector to mark the end of each string. Many studies nowadays use string collections in experiments without turning to dedicated tools for multi-string BWT, e.g. (Bannai *et al.* 2020, Kuhnle *et al.* 2020, Puglisi and Zhukova 2021); often the input strings are turned into one single sequence using one of the methods described above, and then the single-string BWT is computed; it is, however, not always stated explicitly which was the method used to obtain one sequence. Underlying this is the implicit assumption that all methods are equivalent.

In 2007, Mantaci *et al.* (2007) introduced the *extended Burrows–Wheeler transform* (eBWT), which generalizes the BWT to a multiset of strings. The eBWT, like the BWT, is reversible, and maintains other properties of the BWT such as fast pattern matching functionality. The eBWT can handle both linear and circular strings and is thus particularly well applicable in bioinformatics, since many genomic sequences are circular, including mitochondrial DNA, bacterial, and some viral DNA (Boucher *et al.* 2024).

Since then, however, the term “extended BWT” has come to be used as a generic term to denote the BWT of a collection of sequences. This is unfortunate, as the eBWT has several properties, such as independence from the input order, which the other methods do not share; and it is defined using a different order relation from the classical BWT (see Section 2).

Two of the tools listed compute the eBWT according to the original definition, pfpebwt and cais, both (Boucher *et al.* 2021a). The very recent software lFGSACA (Olbrich *et al.* 2024, in press), which was brought to our attention during the review process, also computes the original eBWT; it is, however, a C++ library rather than a command-line tool. All other tools listed append an end-of-string character to the input strings, explicitly or implicitly, and as a consequence, the resulting transforms differ from the one defined in Mantaci *et al.* (2007). Moreover, the output in most cases depends on the input order of the sequences (except for those tools that compute what we term dolEBWT, colexBWT, or optBWT, see Section 3). The exact nature of this dependence differs from one transform to another. The result is that the BWT variants computed by different tools on the same dataset, or by the same tool on the same dataset but given in a different order, may vary considerably.

### 1.2 Multidollar BWT

The BWT-variant which we term mdolBWT is the most general one, in the sense that all others, except for the eBWT, can be simulated by mdolBWT. This is also the variant output by most tools, and it is dependent on the input order: both the transform itself and the number of runs varies depending on the order in which the strings are concatenated. Bentley, Gibney, and Thankachan recently gave a linear-time algorithm for computing mdolBWT with the minimum number of runs amongst all input string orders (Bentley *et al.* 2020). (The same paper includes an NP-hardness result regarding finding the best order on the alphabet, which is a different problem.) In order to study the variation of the parameter *r*, we first implemented a variant of this algorithm counting the minimal number of runs, which later led to a new tool, optimalBWT, computing the optBWT (Cenzato *et al.* 2023). We use this tool in our experiments as a baseline for the number of runs of the other BWT-variants. On our real-life biological datasets, the parameter *r* varies by up to a multiplicative factor of 4.2 between the different variants. It was shown in Cenzato *et al.* (2023) that an improvement by a multiplicative factor of up to 31 can be obtained between the input order and optBWT, again on real-life biological datasets.

### 1.3 What is not covered

This paper deals with tools for string collections, so we did not include any tool that computes the BWT of a single string, such as libdivsufsort, sais-lite-lcp, libsais, or bwtdisk (Ferragina *et al.* 2012). Although in many cases, these are the tools used for collections of strings, the transform they compute depends on the method with which the string collection was turned into a single string, as explained above. Nor did we include other BWT variants for single strings such as the bijective BWT (Gil and Scott 2012, Köppl *et al.* 2020), since, again, these were not designed for string collections.

We did not include Big-xBWT (Gagie *et al.* 2021), a tool for compressing and indexing read collections, which computes the xBWT of Ferragina *et al.* (2005, 2009), and requires a reference sequence in addition to the string collection. The xBWT is not necessarily a permutation of the input characters. Nor is the tool (Ohlebusch *et al.* 2018) for reference-free xBWT included in this review: even though it does not require a reference sequence, it, too, computes the xBWT and not the BWT. Finally, we did not include (Cazaux and Rivals 2019), since its method for concatenating the input strings (using the same separator symbol but without an additional end-of-string character) has not been implemented.

### 1.4 Our contributions

1. We identify six distinct BWT variants which are computed by 18 publicly available tools, specifically designed for string collections. We formally describe the differences between these, identifying specific intervals to which differences are restricted.
2. We describe the impact on the number *r* of runs of the BWT and give an upper bound on how much the colexicographic order (sometimes referred to as “reverse lexicographic order”) can differ from the optimal order of Bentley *et al.* (2020).
3. We complement our theoretical analysis with extensive experiments, comparing the BWT variants on eight real-life datasets with different characteristics.
4. We suggest a way of standardizing the parameter *r*, thus showing how to eliminate the ambiguity caused by the presence of different BWT-variants.

To the best of our knowledge, this is the first systematic study of the different BWT variants in use for collections of strings. In the following, we give the necessary definitions in Section 2. In Section 3, we present the BWT variants and analyze their differences; we discuss the effects on the repetitiveness measure *r* in Section 4. A summary of our experimental results is given in Section 5. We draw some conclusions from our study in Section 6. Proofs, details of the experimental setup, along with the full tables with detailed results on all eight datasets are included in the Supplementary Material. Source code and scripts to replicate the results and download the data used in the article are available at https://github.com/davidecenzato/BWT-variants-for-string-collections.

A preliminary version of this article appeared in Cenzato and Lipták (2022).

## 2 Preliminaries

Let Σ be a finite ordered alphabet of size *σ*. We use the notation T=T[1..n] for a string *T* of length *n* over Σ, T[i] for the *i*th character, and T[i..j] for the substring T[i]⋯T[j] of *T*, where i ≤ j; the length of *T* is denoted |T|, and the empty string is denoted *ε*. For a string *T* over Σ and an integer *m *>* *0, we write *T ^m^* for the *m*-fold concatenation of *T*. A string *T* is called *primitive* if $T=U^{m}$ implies *T *=* U* and *m *=* *1. Every string *T* can be written uniquely as $T=U^{m}$, where *U* is primitive; in this case, we refer to *U* as root(T) and to *m* as exp(T). In other words, for every string *T* it holds that $T=\text{root}{\left(T\right)}^{\;\text{exp}(T)}$. Often, an end-of-string character (usually denoted $) is appended to the end of *T*; this character is not an element of Σ and is smaller than all characters from Σ. Note that appending a $ makes any string primitive.

String *S* is a *conjugate* of string *T* if S=T[i..n]T[1..i−1] for some i∈{1,…,n} (also called the *ith rotation* of *T*). It is easy to see that a string of length *n* has *n* distinct conjugates if and only if it is primitive. A *run* in string *T* is a maximal substring consisting of the same character; we denote by runs(T) the number of runs of *T*. For example, runs(CAAGGGA)=4.

For two strings *S*,*T*, the *(unit-cost) edit distance*, or *Levenshtein distance*, ${\text{dist}}_{\text{edit}}(S,T)$ is defined as the minimum number of operations necessary to transform *S* into *T*, where an operation can be deletion or insertion of a character, or substitution of a character by another. The *Hamming distance* ${\text{dist}}_{H}(S,T)$, defined only if |S|=|T|, is the number of positions *i* such that S[i]≠T[i]. The *lexicographic order* on $\Sigma^{*}$ is defined as follows: $S{<}_{\text{lex}}T$ if *S* is a proper prefix of *T*, or if there exists an index *j* s.t. S[j]<T[j] and for all *i *<* j*, S[i]=T[i]. The *colexicographic order*, or *colex-order* (referred to as *reverse lexicographic order* or *rlo* in Li (2014) and Cox *et al.* (2012)), is defined as follows: $S{<}_{\text{colex}}T$ if $S^{\text{rev}}{<}_{\text{lex}}T^{\text{rev}}$, where $X^{\text{rev}}=X[n]X[n-1]\cdots X[1]$ denotes the reverse of the string X=X[1..n].

For a string T=T[1..n] over Σ, the BWT (Burrows and Wheeler 1994), BWT(T), is a permutation of the characters of *T*, given by concatenating the last characters of the lexicographically sorted conjugates of *T*. In Table 2, we give two examples: BWT(CAGAGA)=GGCAAA, and BWT(CAGAGA$)=AGGC$AA.

**Table 2.** BWT of the strings CAGAGA and CAGAGA$, and eBWTof the string collections {GTC, GT}, and {GTC$,GT$}.

CAGAGA	BWT	CAGAGA$	BWT	{GTC, GT}	eBWT	{GTC$,GT$}	eBWT
ACAGAG	G	$CAGAGA	A	CGT	T	$GT	T
AGACAG	G	A$CAGAG	G	GTC	C	$GTC	C
AGAGAC	C	AGA$CAG	G	GT	T	C$GT	T
CAGAGA	A	AGAGA$C	C	TCG	G	GT$	$
GACAGA	A	CAGAGA$	$	TG	G	GTC$	$
GAGACA	A	GA$CAGA	A			T$G	G
		GAGA$CA	A			TC$G	G

It follows from the definition of the BWT that two strings *S*, *T* are conjugates if and only if BWT(S)=BWT(T). Indeed, the BWT is reversible up to conjugates: if a string *L* is the BWT of some string *T*, then a string *S* can be computed in linear time such that L=BWT(S), and thus *S* is a conjugate of *T*. To make the BWT uniquely reversible, one can add an index to it, marking the lexicographic rank of the conjugate in input. For example, BWT(CAGAGA)=GGCAAA, and the index 4 specifies that the input was the 4th conjugate in lexicographic order. Alternatively, one adds a $ to the end of *T*, which makes the input unique: BWT(CAGAGA$) = AGGC$AA, and CAGAGA$ is the only string ending in $ with this BWT. Note on the example that BWT with and without end-of-string symbol can be quite different.

An important parameter of the BWT of string *T* is the number of runs r(T)=runs(BWT(T)). It is well-known that on repetitive inputs, the BWT tends to produce long runs of the same character, making it amenable to compression via runlength-encoding (RLE). In our example, r(CAGAGA)=3, while the original string has 6 runs. This property, referred to as the *clustering effect* of the BWT, is taken advantage of by compressed data structures such as the RLFM-index (Mäkinen and Navarro 2005) or the *r*-index (Gagie *et al.* 2020).

Next we define the *omega-order* (Mantaci *et al.* 2007) on $\Sigma^{*}$: $S{≺}_{\omega }\;T$ if root(S)=root(T) and exp(S)<exp(T), or if $S^{\omega }{<}_{\text{lex}}\;T^{\omega }$ (implying root(S)≠root(T)), where $T^{\omega }$ denotes the infinite string obtained by concatenating *T* infinitely many times. The omega-order relation coincides with the lexicographic order if neither of the two strings is a proper prefix of the other. The two orders can differ otherwise, e.g. $\mathrm{GT}{<}_{\text{lex}}\;\mathrm{GTC}$ but $\mathrm{GTC}{≺}_{\omega }\;\mathrm{GT}$.

For a multiset of strings $M=\{T_{1},\ldots ,T_{k}\}$, the eBWT, eBWT(M) (Mantaci *et al.* 2007), is a permutation of the characters of the strings in M, given by concatenating the last characters of the conjugates of each *T_i_*, for i=1,…,k, listed in omega-order. For example, the omega-sorted conjugates of M={GTC,GT} are: CGT,GTC,GT,TCG,TG, hence, eBWT(M)=TCTGG, see Table 2. Again, adding the indices of the input conjugates, in this case 2 and 3, makes the eBWT reversible, see Mantaci *et al.* (2007) for details.

## 3 BWT variants for string collections

We identified six distinct transforms, listed below, which were computed by the tools given in Table 1. Let $M=\{T_{1},\ldots ,T_{k}\}$ be a multiset of strings, with total length $N_{M}=\sum_{i=1}^{k}|T_{i}|$. Since several of the data structures depend on the order in which the strings are listed, we implicitly regard M as a list $\left[T_{1},\ldots ,T_{k}\right]$, and write ρ(M) for a specific input order *ρ*.

1. **extended BWT:** eBWT(M) of Mantaci *et al.* (2007) (see Section 2)
2. **dollar-eBWT:** $\text{dolEBWT}(M)=\text{eBWT}(\{T_{i}\$|T_{i}\in M\})$

3. **multidollar BWT:** $\text{mdolBWT}(M)=\text{BWT}(T_{1}{\$}_{1}T_{2}{\$}_{2}\cdots T_{k}{\$}_{k})$ , where dollars are assumed to be smaller than characters from Σ and ${\$}_{1}<{\$}_{2}<\ldots <{\$}_{k}$
4. **colexicographic BWT:** colexBWT(M)=mdolBWT(γ(M)) , where *γ* is the colexicographic (“reverse lexicographic,” rlo) order of the strings in M.
5. **optimal BWT:** optBWT(M)=mdolBWT(opt(M)) , where opt(M) is the order given by the algorithm of Bentley *et al.* (2020), which minimizes the number of runs (see Section 4 for details).
6. **concatenated BWT:** $\text{concBWT}(M)=\text{BWT}(T_{1}\$T_{2}\$\cdots T_{k}\$\#)$ , where #<$.

Because all BWT variants except the eBWT use additional end-of-string symbols as string separators, we refer to these by the collective term *separator-based BWT variants*. In Table 1, we show the six transforms on our running example of five DNA-strings, and give first properties of these transforms. For ease of exposition and comparison, we replaced all separator-symbols by the same dollar-sign $, even where, conceptually or concretely, different dollar-signs are assumed to terminate the individual strings. This is the case for mdolBWT and its special cases, colexBWT and optBWT. Moreover, the concBWT contains one additional character, the final end-of-string symbol, here denoted by #, which is smaller than all other characters; thus, the additional rotation starting with # is the smallest and results in an additional dollar in the first position of the transform. To facilitate the comparison with the other transforms, we remove this first symbol from concBWT and replace the # by $.

It is important to point out that the programs listed in Table 1 do not necessarily use the definitions given here; however, in each case, the resulting transform is the one claimed, up to renaming or removing separator characters, see Sections 3.1 and 3.2.

### 3.1 The effect of adding separator symbols

The first obvious difference between the eBWT and the separator-based variants is their length: eBWT(M) has length $N_{M}$, while all other variants have length $N_{M}+k$, since they contain an additional character (the separator) for each input string.

In all separator-based transforms, the k-length prefix consists of a permutation of the last characters of the input strings. This is because the rotations starting with the dollars are the first k lexicographically. On the other hand, in the eBWT, these k characters occur interspersed with the rest of the transform; namely, in the positions corresponding to the omega-ranks of the input strings $T_{i}$ (see Tables 1 and 3).

**Table 3.** From left to right we show the eBWT, the dolEBWT, the mdolBWT, the colexBWT, the optBWT, and the concBWT of the string collection M={ATATG,TGA,ACG,ATCA,GGA}.^a^

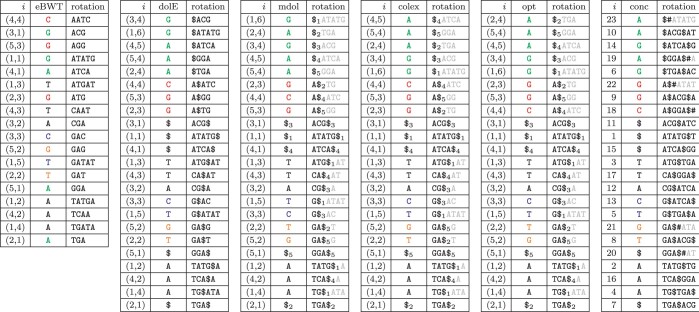

a Indices are given with reference to the numbering $T_{1}=\mathrm{ATATG},T_{2}=\mathrm{TGA},T_{3}=\mathrm{ACG},T_{4}=\mathrm{ATCA},T_{5}=\mathrm{GGA}$. Note that we give the rotations according to Lemma 1.

In general, adding a $ to the end of the strings introduces a distinction, not present in the eBWT, between suffixes and other substrings: since the separators are smaller than all other characters, occurrences of a substring as suffix will be listed en bloc before all other occurrences of the same substring, while in the eBWT, these occurrences are listed interspersed with the other occurrences of the same substring.

Example 1. Let M={AACGAC, TCAC} and *U* = AC. *U* occurs both as a suffix and as an internal factor; the characters preceding it are A (internal substring) and C,G (suffix), and we have eBWT(M)=CGACATAACC, dolEBWT(M)=CC$GCAAATAC$.

Finally, it should be noted that adding end-of-string symbols to the input strings changes the definition of the order applied. As observed above, the omega-order coincides with the lexicographic order on all pairs of strings S,T where neither is a proper prefix of the other; but with end-of-strings characters, no input string can be a proper prefix of another. Thus, on rotations of the $T_{i}\$$’s, the omega-order equals the lexicographic order. As an example, consider the multiset M={GTC$, GT$} from Section 2: we have the following omega-order among the rotations: $GT, $GTC, C$GT, GT$, GTC$, T$G, TC$G (see Table 2), which coincides with the lexicographic order. Similarly, adding *different* dollars $_1_, $_2_, …, $_*k*_ and applying the omega-order results again in the lexicographic order between the rotations, with different dollar symbols considered as distinct characters. This implies:

Lemma 1. *Let* $M=\{T_{1},T_{2},\ldots ,T_{k}\}$*be a string collection. Then*

1.
dolEBWT(M)=mdolBWT(lex(M)) *, where* lex(M)*denotes the lexicographic order of the strings in* M*;*
2.
$\text{mdolBWT}(M)=\text{eBWT}(\{T_{i}{\$}_{i}|i=1,\ldots ,k\})$ *, up to renaming of dollars.*

Regarding the differences among the separator-based BWT variants, we will show that all differences occur in certain well-defined intervals of the BWT, and that the differences themselves depend only on a specific permutation of {1,…,k}, given by the combination of the input order, the lexicographic order of the input strings, and the BWT variant applied. In Table 3, we give the full BWT matrices for all separator-based BWT variants.

### 3.2 Interesting intervals

Let us call a string *U* a *shared suffix* w.r.t. multiset M if it is the suffix of at least two strings in M. Let *b* be the lexicographic rank of the smallest rotation beginning with U$ and e the lexicographic rank of the largest rotation beginning with U$, among all rotations of strings T$, where T∈M. (One can think of [b,e] as the suffix-array interval of U$.) We call [b,e] an *interesting interval* if there exist i≠j s.t. *U* is a suffix of both *T_i_* and *T_j_*, and the preceding characters in *T_i_* and *T_j_* are different, i.e. the two occurrences of *U* as suffix of *T_i_* and *T_j_* constitute a left-maximal repeat. (Put in different terms, interesting intervals correspond to internal nodes in the suffix tree of the reverse string, within the subtree of $.) Clearly, [1,k] is an interesting interval unless all strings end with the same character. Note that interesting intervals differ both from the *SAP-intervals* of Cox *et al.* (2012) and from the *tuples* of Bentley *et al.* (2020) [called *maximal row ranges* in Manzini (2016)]: the former are the intervals corresponding to *all* shared suffixes U, even if not left-maximal, while the latter include also suffixes U that are not shared.

Lemma 2. *Any two distinct interesting intervals are disjoint.*

We can now narrow down the differences between any two separator-based BWTs of the same multiset. The next proposition states that these can only occur in interesting intervals (part 1). This implies that the dollar-symbols appear in the same positions in all separator-based variants except for one very specific case (part 2). Moreover, we get an upper bound on the Hamming distance between two separator-based BWTs (part 3).

Proposition 1. *Let* $L_{1}\;\mathrm{and}\;L_{2}$*be two separator-based BWTs of the same multiset* M.

1. *If* $L_{1}[i]\neq L_{2}[i]\;\;\mathrm{then}\;\;i\in [b,e]$*for some interesting interval* [b,e].
2. *Let* $I_{1}$*resp.* $I_{2}$*be the positions of the dollars in* $L_{1}$*resp.* $L_{2}$*. If* $I_{1}\neq I_{2}$*then there are* i≠j*such that* $T_{i}$*is a proper suffix of* $T_{j}$.
3.
${\text{dist}}_{H}(L_{1},L_{2})\leq \sum_{[b,e]\;\text{interesting}}\left(e-b+1\right)$ .

Proposition 1 implies that the variation of the different transforms can be explained based solely on what rule is used to break ties for shared suffixes. We will see next how the different BWT variants determine this tie-breaking rule.

## 4 Effects on the parameter *r*

What is the effect of the differences in the BWT variants on the number of runs of the BWT? As the following example shows, the number of runs can differ significantly between different variants.

Example 2. Let M={AAAA,AGCA,GCAA,GTCA,CAAA,CGCA,TCAA,TTCA}. Then there are 28 runs in mdolBWT(M)=

AAAAAAAAACACACACACACAC$GTGTGT$AC$GT$; colexBWT(M)=AAAAAAAAAAAACCCCAACCAC$GGTTGT$AC$GT$ has 18 runs.

The results of Section 3 give us a method to measure the degree to which the BWT variants can differ.

Lemma 3. *Let* [b,e]*be an interesting interval, and*$\left(n_{1},\ldots ,n_{\sigma }\right)$*the Parikh vector of* L[b..e]*, i.e.* $n_{i}$*is the number of occurrences of the* i*th character. Let a be such that* $n_{a}=\max_{i}n_{i}$*, and* $N_{a}=(e-b+1)-n_{a}$*, the sum of the other character multiplicities. Then the maximum number of runs in interval*$[b,e]\mathrm{is e}-b+1\;if\;n_{a}-1\leq N_{a}$*, and* $2N_{a}+1$*otherwise.*

Definition 1. Let M be a multiset and var([b,e]) be the bound on number of runs in [b,e] from Lemma 3. The *variability* of M is

$$\mathrm{var}(M)=\frac{\sum_{[b,e]\;\text{interesting}}var([b,e])}{\sum_{[b,e]\;\text{interesting}}\left(e-b+1\right)}.$$

The colexBWT has been shown experimentally to yield a low number of runs of the BWT (Cox *et al.* 2012, Li 2014). This is because it groups the characters in the interesting intervals in at most σ runs. Even though it does not always minimize r, we can bound its distance from the optimum.

Proposition 2. *Let*L*be the colexBWT of multiset*M*, and let*$r_{\text{OPT}}$*denote the minimum number of runs of any separator-based BWT of*M*. Then*$\text{runs}(L)\leq r_{\text{OPT}}+2\cdot c_{M}$*, where* $c_{M}$*is the number of interesting intervals.*

The algorithm of Bentley *et al.* (2020) for the optimal order for mdolBWT is based on the idea of starting from the colex-order and then adjusting, where possible, the order of the runs within interesting intervals in order to minimize character changes at the borders, i.e. such that the first and the last run of each interesting interval is identical to the run preceding and following that interesting interval. This is equivalent to sorting groups of sequences sharing the same left-maximal suffix. This sorting can be done on each interesting interval independently without affecting the other interesting intervals. In Table 3, we show the result on our toy example, where it reduces the number of runs by 2 w.r.t. colex order. In the next section, we compare the number of runs of the non-separator based BWT variants to the optimum.

## 5 Experimental results

We computed the five BWT variants eBWT, dolEBWT, mdolBWT, concBWT, and colexBWT, on eight different genomic datasets. We used the tool optimalBWT to compute the minimum number of runs (i.e. that of optBWT) and used this as a baseline for comparison with the r parameter of the other BWT-variants. For mdolBWT and concBWT, we used the default input order in which the dataset was downloaded. The eight datasets have different characteristics: four contain short reads [SARS-CoV-2 short (Starr *et al.* 2020), Simons Diversity reads (Mallick *et al.* 2016), 16S rRNA short (Winand *et al.* 2019), Influenza A reads (Van den Hoecke *et al.* 2015)], and four long sequences [SARS-CoV-2 long (Greaney *et al.* 2022), 16S rRNA long (Edgar 2018), Candida auris reads (Woodworth *et al.* 2019), SARS-CoV-2 genomes (Boucher *et al.* 2021a)], with the last being whole viral genomes.

On each of the datasets, we computed the pairwise Hamming distance between separator-based BWTs. To compare them to the eBWT, we computed the pairwise edit distance on a small subset of the sequences (for computational reasons), and the Hamming distance on the small set, for comparison. We generated the following statistics on each of the datasets: number of interesting intervals, fraction of positions within interesting intervals (total length of interesting intervals divided by total length of the dataset), and the dataset’s variability (Definition 1).

In Table 4, we give a brief summary of the experimental results, including these statistics on all eight datasets, as well as the average pairwise distance and average runlength over the five separator-based BWT variants; for full results, see the Supplementary Material.

**Table 4.** Summary of the results on the eight datasets.^a^

Dataset	No. seq	Avg. length	Ratio positions in int. intervals	Variability	Avg. Hamming d. betw. $-sep. BWTs	max *n*/*r*	min *n*/*r*	*n*/*r* optimal
SARS-CoV-2 short	500 000	50	0.792	0.210	0.11754	31.524	7.494	35.125
Simons Diversity reads	500 000	100	0.107	0.976	0.07195	7.873	5.299	8.133
16S rRNA short	500 000	152	0.741	0.058	0.02982	44.253	18.836	44.873
Influenza A reads	500 000	231	0.103	0.363	0.02609	49.172	23.100	50.275
SARS-CoV-2 long	50 000	1075	0.175	0.037	0.00464	73.204	57.568	74.498
16S rRNA long	16 741	1502	0.047	0.104	0.00289	46.879	45.015	47.140
Candida auris reads	50 000	2483	0.007	0.497	0.00246	1.732	1.726	1.732
SARS-CoV-2 genomes	2000	29 805	0.001	0.148	0.00012	521.610	499.549	523.240

a From left to right we report dataset names, number of sequences and average sequence length, the ratio of positions in interesting intervals, the variability of the dataset (Definition 1), the average normalized Hamming distance between any two of the separator-based BWT variants (optBWT not included). In the last column, we report the average runlength of the optBWT, and in the previous two columns, the maximum and minimum average runlength (n/r) taken over the other five BWT variants.

**Figure 1.**
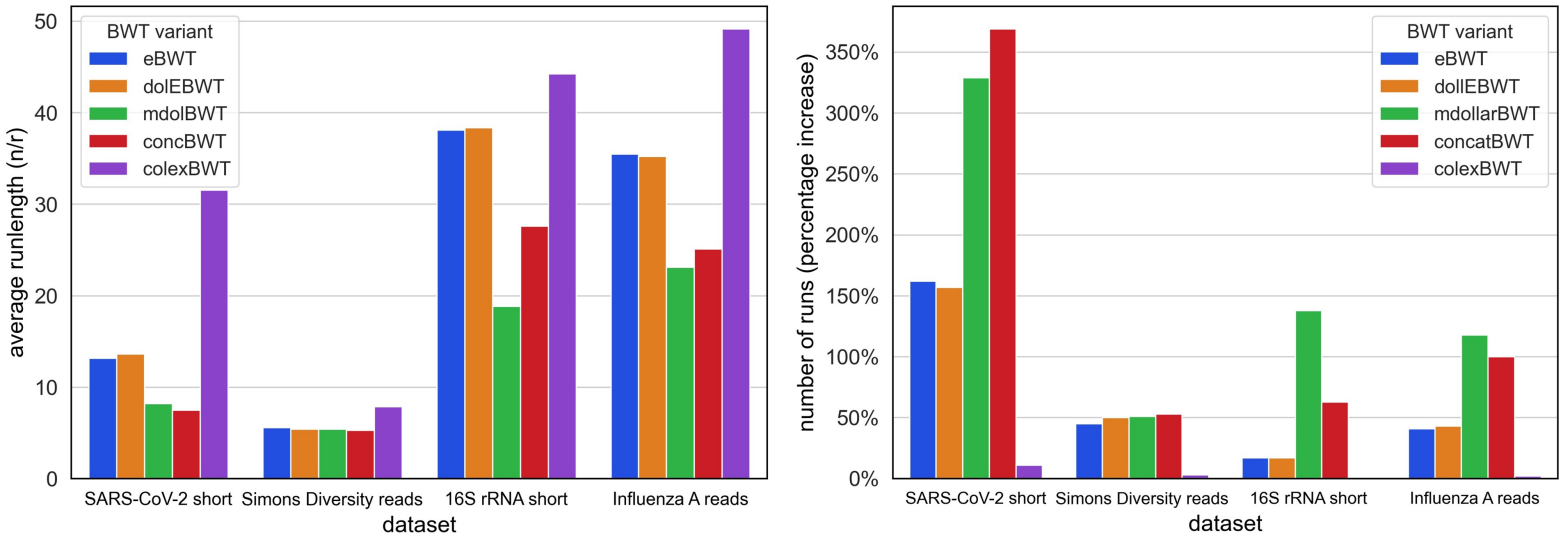
Results regarding *r* on short sequence datasets, of all BWT variants. Left: average runlength (*n*/*r*). Right: number of runs (percentage increase with respect to optBWT).

**Figure 2.**
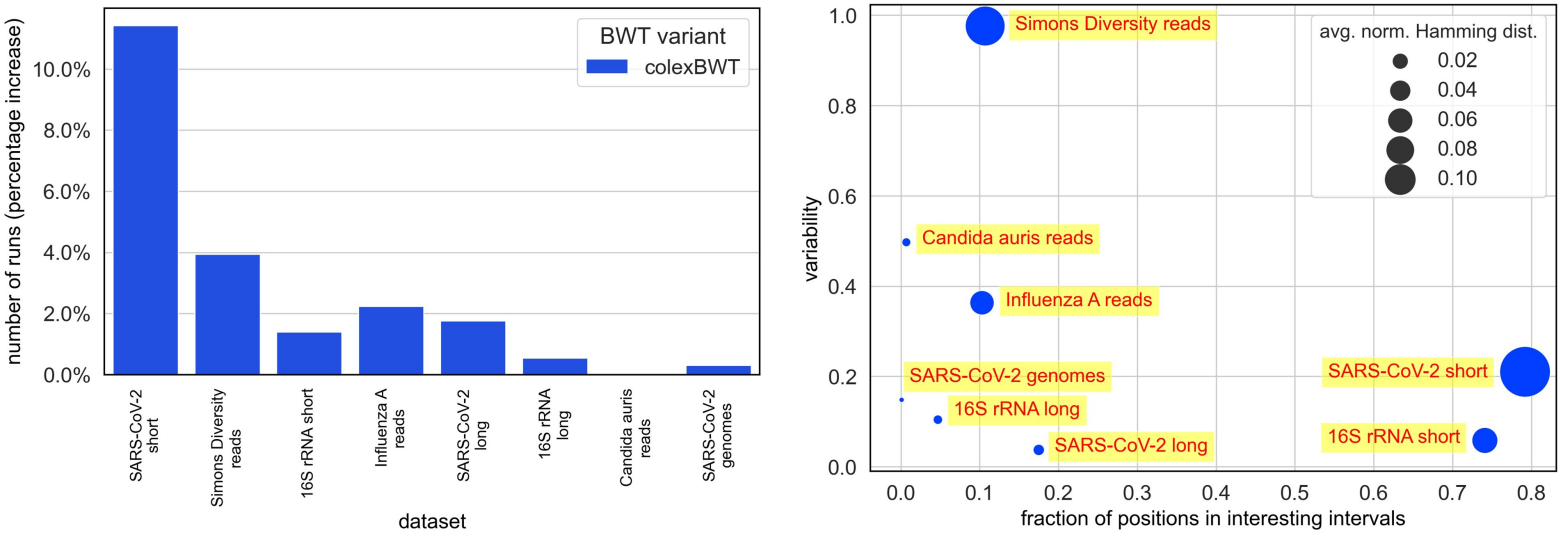
Left: number of runs of the colexBWT with respect to optimal BWT (percentage increase) on all eight datasets. Right: average normalized Hamming distance variations with respect to variability and fraction of positions in interesting intervals on all datasets.

The experiments showed a high variation in the number of runs in particular on datasets of short sequences. The highest difference was between colexBWT and concBWT, by a multiplicative factor of over 4.2, on the SARS-CoV-2 short dataset. In Fig. 1, we plot the average runlength n/r for the four short sequence datasets, and the percentage increase of the number of runs w.r.t. $r_{\mathrm{opt}}$. The variation is less pronounced on the one dataset which is less repetitive, namely Simons Diversity reads. Recall that the mdolBWT and concBWT vary depending on the input permutation. On most long sequence datasets, on the other hand, the differences were quite small (see Supplementary Material). To better understand how far the colexBWT is from the optimum w.r.t. the number of runs, we plot in Fig. 2 (left) the number of runs of colexBWT w.r.t. to $r_{\mathrm{opt}}$, on all eight datasets. The strongest increase is on short sequences, where the variation among all BWT variants is high, as well; on the long sequence datasets, with the exception of SARS-CoV-2 long sequences, the colexBWT is very close to the optimum; however, note that on those datasets, all BWTs are close to the optimum.

The average number of runs and the average pairwise Hamming distance strongly depend on the length of the sequences. If the collection has a lot of short sequences which are very similar, then the differences between the BWTs both w.r.t. the number of runs, and as measured by the Hamming distance, can be large. This is because there are a lot of maximal shared suffixes and so, many positions are in interesting intervals. To better understand this relationship, we plotted, in Fig. 2 (right), the average Hamming distance against the two parameters variability and fraction of positions in interesting intervals. We see that the two datasets with highest average Hamming distance have at least one of the two values very close to 1, while for those datasets where both values are very low, the BWT variants do not differ very much.

The input order used by the mdolBWT and the concBWT is the order in which the input sequences appear when the dataset is downloaded. Our study shows that only a few input permutations can minimize the number of runs of the resulting BWT, namely those orders that group the characters inside the interesting intervals in at most σ runs, such as the order of Bentley *et al.* and the colexicographic order. Since there are k! possible input permutations, selecting an arbitrary input order will likely result in a BWT whose number of runs is much larger than the optimal one, especially on datasets with high variability.

## 6 Conclusion

We presented the first study of the different variants of the BWT for string collections. We found that the transforms computed by different tools differ not insignificantly, as measured by the pairwise Hamming distance: up to 12% between different BWT variants on the same dataset in our experiments. We showed that most current tools implement BWT variants that are input order dependent, so that the same tool can produce different outputs if the input set is permuted. These differences extend also to the number of runs r, a parameter that is central in the analysis of BWT-based data structures, and which is increasingly being used as a measure of the repetitiveness of the dataset itself.

With string collections replacing individual sequences as the prime object of research and analysis, and thus becoming the standard input for text indexing algorithms, we believe that it is all the more important for users and researchers to be aware that not all methods are equivalent, and to understand the precise nature of the BWT variant produced by a particular tool.

We suggest further to standardize the definition of the parameter r for string collections, using either the colexicographic order—implemented by the tool ropebwt2 (Li 2014)—or the optimal order of Bentley *et al.* (2020)—implemented by the tool optimalBWT (Cenzato *et al.* 2023). In this paper, we found that the number of runs can vary by up to a factor of 4.2 on real-life biological datasets, while in Cenzato *et al.* (2023), a factor of 31 was shown on other biological data. Not only does this heavily impact the space requirements of BWT-based data structures, but it also means that using the average runlength n/r as a repetitiveness measure of a dataset is ambiguous, unless the research community agrees on the BWT variant being used for the definition of this parameter.

## Supplementary Material

btae333_Supplementary_Data
