# Supplementary material for: A survey of BWT variants for string collections
Source: Bioinformatics. 2024 May 24;40(7):btae333. doi: 10.1093/bioinformatics/btae333 (PMC11286279; doi:10.1093/bioinformatics/btae333)
Supplement: btae333_Supplementary_Data [file btae333_supplementary_data.pdf]

Bioinformatics, 2023, 1–3
doi: DOI HERE
Advance Access Publication Date: Day Month Year
Article

Supplementary Material

A survey of BWT variants for string collections

Davide Cenzato[1] and Zsuzsanna Lipták[2,*]

[1]Department of Environmental Sciences, Informatics and Statistics, Ca' Foscari University, Venice, Italy and
[2]Department of Computer Science, University of Verona, Verona, Italy

*Corresponding author. zsuzsanna.liptak@univr.it

FOR PUBLISHER ONLY Received on Date Month Year; revised on Date Month Year; accepted on Date Month Year

A. Missing proofs

Proof of Lemma 1 For *1.*, note that the order of the strings $T_i\$$ is the lexicographic order, since $T_i\$ <_\omega T_j\$$ if and only if $T_i\$ <_{\text{lex}} T_j\$$ if and only if $T_i <_{\text{lex}} T_j$. For *2.*, consider that no two rotations can be one prefix of the other, resulting in the lexicographic order between rotations, with the dollar-signs breaking ties. The only difference between the two transforms is now that for $i > 1$, $\$_i$ in the right transform is replaced by $\$_{i-1}$ in the left transform, and $\$_1$ is replaced by $\$_k$. □

Proof of Lemma 2 Follows from the fact that no two distinct substrings ending in \$ can be one prefix of the other. □

Proof of Proposition 1 *1.* Let $L_1[i] = \mathtt{x}$ and $L_2[i] = \mathtt{y}$. Since all separator-based BWT variants use the lexicographical order of the rotations, this means that there exists a substring U which is preceded by x in one string T_j and by y in another $T_{j'}$, the first occurrence has rank i in one BWT and the other has rank i in the other BWT variant. This implies that the two occurrences are followed by two dollars, and either the two dollars are different, or they are the same dollar, and the subsequent substrings are different. Therefore, U defines an interesting interval. Parts *2.* and *3.* follow from *1.* □

Proof of Lemma 3 Place the $n_{\mathtt{a}}$ a-characters in a row, creating $n_{\mathtt{a}} + 1$ gaps, namely one between each adjacent a, and one each at the beginning and at the end. Now place all b-characters, each in a different gap; since $n_{\mathtt{a}}$ is maximum, there are enough gaps. Then place all c's, first filling gaps that are still empty, if any, then into gaps without c, etc. We never have to place two identical characters in the same gap. If the total number of non-a-characters is at least than $n_{\mathtt{a}} - 1$, then we can fill every gap, thus separating all a's, and creating a run for every character of I. If we have fewer than $n_{\mathtt{a}} - 1$ characters, then we are still creating two runs with each non-a-character, but we cannot separate all a's. □

Proof of Proposition 2 Let $I = [b_I, e_I]$ be an interesting interval containing d distinct characters, and let U be the shared suffix defining I. Since the strings are listed according to the colex order, all strings in which U is preceded by the same character will appear in one block, and therefore, L has exactly d runs in the interval I. Let $L_{b_I-1} = \mathtt{x}$ and $L_{e_I+1} = \mathtt{y}$. If x occurs in I and it is not the first run of I (i.e., $L_{b_I} \neq \mathtt{x}$), then listing first the strings where U is preceded by x would reduce the number of runs by 1; similarly, listing those where y precedes U as last of the group would reduce the number of runs by 1. By Prop. 1, this is the only possibility for varying the number of runs. □

B. Experimental setup

All datasets are stored in FASTA format.

We used three tools for computing the five BWT variants; `pfpebwt`, `ropebwt2` and `Big-BWT`. In order to make the BWTs comparable we did some adaptations to both tools and inputs. We modified `ropebwt2` to make it work with the same character order as the other tools, i.e. $\$ < \mathtt{A} < \mathtt{C} < \mathtt{G} < \mathtt{N} < \mathtt{T}$. Then we used `ropebwt2` for computing both the mdolBWT and the colexBWT using the `-R` and `-R -s` flags respectively. We used `pfpebwt` for constructing both the eBWT and the dolEBWT variants. In order to compute the dolEBWT, we modified the input files, appending an end-of-string character at the end of each sequence. Finally, for computing the concBWT, we removed the headers from the FASTA files, arranging the sequences in newline separated files, and ran `Big-BWT` without additional flags on these newline separated files.

C. Further information on the tools

We tested all 18 tools extensively, and determined which data structure they compute, using both our tests and the algorithm descriptions in the respective papers. In this section, we include further information about some of these tools.

- `pfpebwt` is a tool computing the eBWT of string collections (`https://github.com/davidecenzato/PFP-eBWT.git`). It takes in input a fasta file and gives in output the eBWT in either

© The Author 2023. Published by Oxford University Press. All rights reserved. For permissions, please e-mail: journals.permissions@oup.com

dataset	no. seq	total length	avg	min	max	n/r (opt)
SARS-CoV-2 short	500,000	25,000,000	50	50	50	35.125
Simons Diversity reads	500,000	50,000,000	100	100	100	8.133
16S rRNA short	500,000	75,929,833	152	69	301	44.873
Influenza A reads	500,000	115,692,842	231	60	251	50.275
SARS-CoV-2 long	50,000	53,726,351	1,075	265	3,355	74.498
16S rRNA long	16,741	25,142,323	1,502	1,430	1,549	47.140
Candida auris reads	50,000	124,150,880	2,483	214	8,791	1.732
SARS-CoV-2 genomes	2,000	59,610,692	29,805	22,871	29,920	523.240

Table 1. Summary of the most important parameters of the eight datasets. From left to right we report the dataset name, the number of sequences, the total length, the average, minimum and maximum sequence length, and the average runlength n/r of the optimum BWT according to Bentley et al. [2].

plain ASCII text or RLE (run-length-encoded) format. We used (a) no flags for long sequences, and (b) the flags `-w 10 -p 10 -n 3 --reads` for short sequences. We included it in two different rows of Table 1 because by default `pfpebwt` computes the eBWT, but it can compute the dolEBWT if the sequences have explicit end-of-string characters (not in multi-thread mode).

- `cais` is a tool implementing the `SAIS_for_eBWT` algorithm [3], which computes both the eBWT and the dolEBWT (`https://github.com/davidecenzato/cais.git`) depending on the input flag. It takes in input a fasta file, a fastq file, or a plain text file and gives in output the final transform in plain ASCII text. The `-c` and `-a` flags enable to output the conjugate array along with the resulting BWT.
- `G2BWT` is a tool computing the dolEBWT of short sequence collections (`https://bitbucket.org/DiegoDiazDominguez/lms_grammar/src/bwt_imp2`). It takes in input newline separated files. Even though it is not stated explicitly, this tool computes the dolEBWT because, when it constructs the grammar, it uses dollars for separating adjacent strings. Thus, also the string rotations will contain dollars. We tested it using the default settings.
- `msbwt` is a tool implementing the Holt and McMillan [4] merge-based BWT construction algorithm (`https://github.com/holtjma/msbwt.git`). It takes in input a list of one or several fastq files. Even if this tool uses the `BCR` approach [1] for computing the BWTs to merge, it actually computes the dolEBWT. This is because it features a preprocessing where it sorts the input strings lexicographically. Thus, the resulting mdolBWT corresponds to the dolEBWT.
- `BEETL` is a suite containing several tools, including a tool computing the mdolBWT of string collections using an implementation of the BCR and BCR-ext algorithms [1] (`https://github.com/BEETL/BEETL.git`). This tool requires that all input sequences have to have the same length. We tested this tool using `--output-format ASCII` and `--concatenate-output` flags. This tool also computes the a BWT variant similar to the colexBWT by using the `--sap-ordering` flag (BCR-ext mode only).
- `BCR_LCP_GSA` is a tool computing the mdolBWT of sting collections in semi-external memory (`https://github.com/giovannarosone/BCR_LCP_GSA`). It implements an algorithm similar to `BCR` contained in the BEETL tool, but it can process a string collection containing sequences of different lengths. It takes in input a fasta file, a fastq file, or a gz-compressed fastq file. It computes the mdolBWT following the method of Bauer et al., described in [1]. We set the `'dataTypeLengthSequences'` variable in `Parameters.h` to 1.
- `ropebwt2` is a tool computing the FM-index and the mdolBWT of string collections (`https://github.com/lh3/ropebwt2.git`), using an approach similar to `BCR`. It takes in input a fasta file, a fastq file, or a gz compressed fastq file. We listed it in two different rows of Table 1because it computes the mdolBWT or the colexBWT, depending on the flags. We used the `-R` and the `-R -s` flags, respectively, to obtain the two transforms. In addition, we modified `main.c` in order to change the order of the characters to `$ < A < C < G < N < T`.
- `merge-BWT` computes the mdolBWT of a string collection by merging the BWTs of subcollections of the input (`https://github.com/jltsiren/bwt-merge.git`). It takes in input a list of one or several mdolBWTs. The order of the dollars will depend on the order in which the input BWTs are listed. We tested it using `-i plain_sorted and -o plain_sorted` flags. We computed the BWTs of the subcollections using `ropebwt2`.
- `nvSetBWT` is a tool included in `nvbio` suite (`https://github.com/NVlabs/nvbio.git`). It takes in input either a fastq or a newline separated file. We tested it using the `-R` flag for skipping the reverse strand. However, even if the algorithmic descriptions in [7, 5] seem to describe the mdolBWT, the output of the current version (version 1.1) does not correspond to a possible BWT because the Parikh vector is different from that of the input.
- `eGSA` computes the generalized enhanced suffix array and the mdolBWT of a string collection (`https://github.com/felipelouza/egsa.git`). It takes in input a text file, a fasta file, or a fastq file. It uses the gSACA-K algorithm for computing the suffix array of subcollections of the input and then merges all suffix arrays. Thus it computes the mdolBWT. We tested it with the `-b` flag.
- `eGAP` computes the mdolBWT, and optionally the LCP-array (longest common prefix array) and DA (document array) of a string collection (`https://github.com/felipelouza/egap.git`). It takes in input a newline separated file, a fasta file, or a fastq file. We tested it with default settings.
- `bwt-lcp-parallel` computes the mdolBWT and the LCP-array of a collection of short sequences (`https://github.com/AlgoLab/bwt-lcp-parallel.git`). It takes in input fasta files and does not support the `N` character. We tested it using standard settings.
- `gsufsort` computes the SA, LCP and mdolBWT of a string collection (`https://github.com/felipelouza/gsufsort.git`), using the gSACA-K algorithm of [6]. It takes in input a newline

separated file, a fasta file, or a fastq file. We tested it using `--fasta` and `--bwt` flags.

- `grlBWT` is a tool computing the mdolBWT of string collections using an induced suffix sorting based algorithm that keeps the intermediate data structures in compressed form (`https://github.com/ddiazdom/grlBWT`). It takes in input a concatenated string collection and gives in output the mdolBWT in run-length compressed form. We tested it with the default parameters and used new-line separated files as input.
- `BigBWT` computes the concBWT, and optionally the suffix array, of a highly repetitive text or string collection (`https://github.com/alshai/Big-BWT.git`) using the Prefix-free parsing (PFP) algorithm. It takes in input a newline separated file or a fasta file. This tool with the `-f` flag is used internally in the r-index implementation (`https://github.com/alshai/r-index`), producing the BWT of the strings concatenated without dollars, thus, the end-of-string symbols have to be added explicitly. On the other hand, the tool without the `-f` flag will compute the BWT of the fasta files without skipping the fasta headers. We used standard parameters and as input newline separated files, the output then is the concBWT.
- `r-pfbwt` is a tool which computes the run-length encoded concBWT by using a similar algorithm than `BigBWT` (`https://github.com/marco-oliva/r-pfbwt`). However, unlike `BigBWT`, `r-pfbwt` employs an improved version of the PFP algorithm, which allows the process of even larger datasets through a recursive pre-processing of the input. We tested it using the `--bwt-only` flag and computed the PFP data structures using the `pfp++` software (`https://github.com/marco-oliva/pfp.git`).
- `CMS-BWT` is a tool computing the concBWT by using the matching statistics to speed up the BWT computation and reduce the memory footprint for large and repetitive datasets (`https://github.com/fmasillo/CMS-BWT.git`). Unlike the other software it requires two input files, one containing a string collection and another containing a reference sequence. It takes in input fasta files and outputs the resulting BWT in plain format or run-length encoding. We tested it using the default parameters.
- `optimalBWT` is a tool computing the optimal BWT of Bentley et al., it features two different construction algorithms, a variant of SAIS of Nong et al. which works in internal memory and a variant of BCR working in semi-external memory (`https://github.com/davidecenzato/optimalBWT.git`). It takes in input either a fasta or fastq file and outputs the resulting BWT in plain ascii text. We tested it using both `-a sais` and `-a bcr` flags.

D. Full results on individual datasets

References

1. M. J. Bauer, A. J. Cox, and G. Rosone. Lightweight algorithms for constructing and inverting the BWT of string collections. *Theor. Comput. Sci.*, 483:134–148, 2013.
2. J. W. Bentley, D. Gibney, and S. V. Thankachan. On the complexity of BWT-runs minimization via alphabet reordering. In *Proc. of 28th Annual European Symposium on Algorithms (ESA 2020)*, volume 173 of *LIPIcs*, pages 15:1–15:13, 2020.
3. C. Boucher, D. Cenzato, Zs. Lipták, M. Rossi, and M. Sciortino. Computing the original eBWT faster, simpler, and with less memory. In *Proc. of 28th International Symposium on String Processing and Information Retrieval (SPIRE 2021)*, volume 12944 of *LNCS*, pages 129–142, 2021.
4. J. Holt and L. McMillan. Merging of multi-string BWTs with applications. *Bioinform.*, 30(24):3524–3531, 2014.
5. C. Liu, R. Luo, and T. W. Lam. GPU-accelerated BWT construction for large collection of short reads. *CoRR*, abs/1401.7457, 2014.
6. F. A. Louza, S. Gog, and G. P. Telles. Inducing enhanced suffix arrays for string collections. *Theor. Comput. Sci.*, 678:22–39, 2017.
7. J. Pantaleoni. BWT of large string sets. *CoRR*, abs/1410.0562, 2014.

SARS-CoV-2 short (500,000 short sequences)

Hamming d. / *norm. Hamming d.*	*Hamming distance on the big dataset*			
	dolEBWT	mdolBWT	concBWT	colexBWT
dolEBWT	0	3,014,183	2,926,602	2,912,860
mdolBWT	0.11820	0	3,013,908	3,102,887
concBWT	0.11477	0.11819	0	3,013,634
colexBWT	0.11423	0.12168	0.11818	0

dataset properties	
no. sequences	500,000
average length	50
total length	25,000,000
no. of interesting intervals	116,598
total length intr.int.s	20,187,840
fraction pos.s in intr.int.s	0.792
variability	0.210

no. runs big dataset		
	r	n/r
eBWT	1,902,148	13.143
dolEBWT	1,868,581	13.647
mdolBWT	3,113,818	8.189
concBWT	3,402,513	7.494
colexBWT	808,906	31.524
optBWT	725,979	35.125

Hamming d. / *norm. Hamming d.*	*Hamming distance on a subset of 5,000 sequences*			
	dolEBWT	mdolBWT	concBWT	colexBWT
dolEBWT	0	21,362	21,196	20,626
mdolBWT	0.08377	0	21,376	21,256
concBWT	0.08312	0.08383	0	21,259
colexBWT	0.08089	0.08336	0.08337	0

small dataset properties	
no. of sequences	5,000
total length	250,000
average length	100
no. of interesting intervals	2,476
total length intr.int.s	180,038
fraction pos.s in intr.int.s	0.706
variability	0.173

edit d. / *norm. edit d.*	*edit distance on a subset of 5,000 sequences*				
	eBWT	dolEBWT	mdolBWT	concBWT	colexBWT
eBWT	0	28,702	43,903	43,828	46,936
dolEBWT	0.11256	0	17,000	16,921	20,104
mdolBWT	0.17217	0.06667	0	16,130	20,812
concBWT	0.17187	0.06636	0.06325	0	20,830
colexBWT	0.18406	0.07884	0.08162	0.08169	0

no. runs small dataset		
	r	n/r
eBWT	52,979	4.719
dolEBWT	50,803	5.019
mdolBWT	54,766	4.656
concBWT	54,698	4.662
colexBWT	37,320	6.833
optBWT	35,904	7.102

Table 2. Results for the SARS-CoV-2 short dataset. First row left: absolute and normalized pairwise Hamming distance between separator-based BWT variants. First row right: summary of the dataset properties. Second row: number of runs and average runlength (n/r) of all BWT variants. Third row left: absolute and normalized pairwise Hamming distance between separator-based BWT variants on a subset of the input collection. Third row right: summary of the dataset properties of a subset of the input collection. Fourth row left: absolute and normalized pairwise edit distance between all BWT variants on a subset of the input collection. Fourth row right: number of runs and average runlength (n/r) of all BWT variants on a subset of the input collection.

Simons Diversity reads (500,000 short sequences)

Hamming d. / norm. Hamming d.	Hamming distance on the big dataset			
	dolEBWT	mdolBWT	concBWT	colexBWT
dolEBWT	0	3,624,283	3,602,362	3,594,438
mdolBWT	0.07249	0	3,628,799	3,623,154
concBWT	0.07133	0.07186	0	3,617,679
colexBWT	0.07189	0.07246	0.07168	0

dataset properties	
no. of sequences	500,000
total length	50,000,000
average length	100
no. of interesting intervals	316,013
total length intr.int.s	5,387,549
fraction pos.s in intr.int.s	0.107
variability	0.976

no. runs big dataset		
	r	n/r
eBWT	8,974,105	5.572
dolEBWT	9,337,122	5.409
mdolBWT	9,362,564	5.394
concBWT	9,530,334	5.299
colexBWT	6,414,356	7.873
optBWT	6,209,567	8.133

Hamming d. / norm. Hamming d.	Hamming distance on a subset of 5,000 sequences			
	dolEBWT	mdolBWT	concBWT	colexBWT
dolEBWT	0	23,742	23,461	23,535
mdolBWT	0.04748	0	23,785	23,722
concBWT	0.04646	0.04710	0	23,660
colexBWT	0.04707	0.04744	0.04685	0

small dataset properties	
no. of sequences	5,000
total length	500,000
average length	100
no. of interesting intervals	3,111
total length intr.int.s	35,404
fraction pos.s in intr.int.s	0.070
variability	0.989

edit d. / norm. edit d.	edit distance on a subset of 5,000 sequences				
	eBWT	dolEBWT	mdolBWT	concBWT	colexBWT
eBWT	0	72,898	72,878	72,918	74,026
dolEBWT	0.14435	0	17,820	17,560	22,481
mdolBWT	0.14431	0.03529	0	17,726	22,586
concBWT	0.14439	0.03477	0.03510	0	22,595
colexBWT	0.14659	0.04452	0.04472	0.04474	0

no. runs small dataset		
	r	n/r
eBWT	77,646	6.439
dolEBWT	81,758	6.177
mdolBWT	81,883	6.167
concBWT	82,779	6.101
colexBWT	64,229	7.862
optBWT	62,117	8.130

Table 3. Results for the Simons Diversity reads dataset. First row left: absolute and normalized pairwise Hamming distance between separator-based BWT variants. First row right: summary of the dataset properties. Second row: number of runs and average runlength (n/r) of all BWT variants. Third row left: absolute and normalized pairwise Hamming distance between separator-based BWT variants on a subset of the input collection. Third row right: summary of the dataset properties of a subset of the input collection. Fourth row left: absolute and normalized pairwise edit distance between all BWT variants on a subset of the input collection. Fourth row right: number of runs and average runlength (n/r) of all BWT variants on a subset of the input collection.

16S rRNA short (500,000 short sequences)

Hamming d. / *norm. Hamming d.*	*Hamming distance on the big dataset*			
	dolEBWT	mdolBWT	concBWT	colexBWT
dolEBWT	0	2,202,008	2,540,310	1,748,072
mdolBWT	0.02881	0	2,201,003	2,202,717
concBWT	0.03324	0.02880	0	2,784,600
colexBWT	0.02287	0.02882	0.03643	0

dataset properties	
no. of sequences	500,000
total length	75,929,833
average length	152
no. of interesting intervals	54,366
total length intr.int.s	56,708,529
fraction pos.s in intr.int.s	0.742
variability	0.058

no. runs big dataset		
	r	n/r
eBWT	1,992,130	38.115
dolEBWT	1,992,211	38.364
mdolBWT	4,057,541	18.836
concBWT	2,767,797	27.614
colexBWT	1,727,127	44.253
optBWT	1,703,234	44.873

Hamming d. / *norm. Hamming d.*	*Hamming distance on a subset of 5,000 sequences*			
	dolEBWT	mdolBWT	concBWT	colexBWT
dolEBWT	0	20,159	23,229	15,835
mdolBWT	0.02635	0	20,024	20,092
concBWT	0.03036	0.02617	0	25,464
colexBWT	0.02070	0.02626	0.03329	0

small dataset properties	
no. of sequences	5,000
total length	765,037
average length	152
no. of interesting intervals	1,376
total length intr.int.s	139,041
fraction pos.s in intr.int.s	0.182
variability	0.222

edit d. / *norm. edit d.*	*edit distance on a subset of 5,000 sequences*				
	eBWT	dolEBWT	mdolBWT	concBWT	colexBWT
eBWT	0	51,683	62,799	63,303	61,732
dolEBWT	0.06756	0	16,968	20,180	14,166
mdolBWT	0.08209	0.02218	0	16,695	19,371
concBWT	0.08274	0.02638	0.02182	0	21,683
colexBWT	0.08069	0.01852	0.02532	0.02834	0

no. runs small dataset		
	r	n/r
eBWT	35,262	21.554
dolEBWT	35,293	21.677
mdolBWT	50,581	15.125
concBWT	38,900	19.667
colexBWT	30,568	25.027
optBWT	30,007	25.495

Table 4. Results for the 16S rRNA short dataset. First row left: absolute and normalized pairwise Hamming distance between separator-based BWT variants. First row right: summary of the dataset properties. Second row: number of runs and average runlength (n/r) of all BWT variants. Third row left: absolute and normalized pairwise Hamming distance between separator-based BWT variants on a subset of the input collection. Third row right: summary of the dataset properties of a subset of the input collection. Fourth row left: absolute and normalized pairwise edit distance between all BWT variants on a subset of the input collection. Fourth row right: number of runs and average runlength (n/r) of all BWT variants on a subset of the input collection.

Influenza A reads (500,000 short sequences)

Hamming d. / *norm. Hamming d.*	dolEBWT	mdolBWT	concBWT	colexBWT
dolEBWT	0	3,040,590	3,038,509	2,938,706
mdolBWT	0.02617	0	3,039,095	3,041,816
concBWT	0.02615	0.02616	0	3,089,670
colexBWT	0.02529	0.02618	0.02659	0

Hamming distance on the big dataset

dataset properties	
no. of sequences	500,000
total length	116,192,842
average length	231
no. of interesting intervals	213,735
total length intr.int.s	11,995,246
fraction pos.s in intr.int.s	0.103
variability	0.363

no. runs big dataset

	r	n/r
eBWT	3,258,605	35.504
dolEBWT	3,298,502	35.226
mdolBWT	5,030,032	23.100
concBWT	4,629,150	25.100
colexBWT	2,362,987	49.172
optBWT	2,311,133	50.275

Hamming distance on a subset of 5,000 sequences

Hamming d. / *norm. Hamming d.*	dolEBWT	mdolBWT	concBWT	colexBWT
dolEBWT	0	23,456	23,456	22,873
mdolBWT	0.02018	0	23,509	23,407
concBWT	0.02018	0.02023	0	24,061
colexBWT	0.01968	0.02014	0.02070	0

small dataset properties	
no. of sequences	5,000
total length	1,162,319
average length	231
no. of interesting intervals	3,062
total length intr.int.s	36,019
fraction pos.s in intr.int.s	0.031
variability	0.966

edit distance on a subset of 5,000 sequences

edit d. / *norm. edit d.*	eBWT	dolEBWT	mdolBWT	concBWT	colexBWT
eBWT	0	75,966	75,935	75,991	76,437
dolEBWT	0.06536	0	18,043	18,316	21,869
mdolBWT	0.06533	0.01552	0	17,835	22,536
concBWT	0.06538	0.01576	0.01534	0	23,078
colexBWT	0.06576	0.01881	0.01939	0.01986	0

no. runs small dataset

	r	n/r
eBWT	81,992	14.115
dolEBWT	85,489	13.596
mdolBWT	89,256	13.022
concBWT	87,867	13.228
colexBWT	70,534	16.479
optBWT	68,900	16.870

Table 5. Results for the Influenza A reads dataset. First row left: absolute and normalized pairwise Hamming distance between separator-based BWT variants. First row right: summary of the dataset properties. Second row: number of runs and average runlength (n/r) of all BWT variants. Third row left: absolute and normalized pairwise Hamming distance between separator-based BWT variants on a subset of the input collection. Third row right: summary of the dataset properties of a subset of the input collection. Fourth row left: absolute and normalized pairwise edit distance between all BWT variants on a subset of the input collection. Fourth row right: number of runs and average runlength (n/r) of all BWT variants on a subset of the input collection.

SARS-CoV-2 long (50,000 long sequences)

Hamming d. / *norm. Hamming d.*	*Hamming distance on the big dataset*			
	dolEBWT	mdolBWT	concBWT	colexBWT
dolEBWT	0	248,189	248,205	255,357
mdolBWT	0.00462	0	248,572	248,631
concBWT	0.00462	0.00462	0	248,765
colexBWT	0.00475	0.00462	0.00463	0

dataset properties	
no. sequences	50,000
total length	53,776,351
average length	1,075
no. of interesting intervals	31,931
total length intr.int.s	9,436,894
fraction pos.s in intr.int.s	0.17548
variability	0.03716

no. runs big dataset		
	r	n/r
eBWT	882,634	60.870
dolEBWT	879,608	61.137
mdolBWT	934,129	57.568
concBWT	934,117	57.569
colexBWT	734,610	73.204
optBWT	721,845	74.498

Hamming d. / *norm. Hamming d.*	*Hamming distance on a subset of 1,500 sequences*			
	dolEBWT	mdolBWT	concBWT	colexBWT
dolEBWT	0	4,936	4,939	5,296
mdolBWT	0.00306	0	4,884	4,908
concBWT	0.00306	0.00303	0	5,012
colexBWT	0.00328	0.00304	0.00310	0

small dataset properties	
no. sequences	1,500
total length	1,612,956
average length	1,075
no. of interesting intervals	1,046
total length intr.int.s	152,035
fraction pos.s in intr.int.s	0.094
variability	0.047

edit d. / *norm. edit d.*	*edit distance on a subset of 1,500 sequences*				
	eBWT	dolEBWT	mdolBWT	concBWT	colexBWT
eBWT	0	19,140	21,809	21,796	22,618
dolEBWT	0.01186	0	4,345	4,322	5,186
mdolBWT	0.01351	0.00269	0	4,110	4,820
concBWT	0.01350	0.00306	0.00255	0	4,893
colexBWT	0.01401	0.00321	0.00299	0.00303	0

no. runs small dataset		
	r	n/r
eBWT	45,262	35.636
dolEBWT	45,155	35.754
mdolBWT	45,572	35.426
concBWT	45,644	35.371
colexBWT	42,516	37.973
optBWT	42,093	38.355

Table 6. Results for the SARS-CoV-2 long dataset. First row left: absolute and normalized pairwise Hamming distance between separator-based BWT variants. First row right: summary of the dataset properties. Second row: number of runs and average runlength (n/r) of all BWT variants. Third row left: absolute and normalized pairwise Hamming distance between separator-based BWT variants on a subset of the input collection. Third row right: summary of the dataset properties of a subset of the input collection. Fourth row left: absolute and normalized pairwise edit distance between all BWT variants on a subset of the input collection. Fourth row right: number of runs and average runlength (n/r) of all BWT variants on a subset of the input collection.

16S rRNA long (16,741 long sequences)

Hamming d. / norm. Hamming d.	Hamming distance on the big dataset			
	dolEBWT	mdolBWT	concBWT	colexBWT
dolEBWT	0	85,960	42,948	67,103
mdolBWT	0.00342	0	85,961	82,890
concBWT	0.00171	0.00342	0	71,264
colexBWT	0.00267	0.00329	0.00283	0

dataset properties	
no. sequences	16,741
total length	25,159,064
average length	1,501
no. of interesting intervals	9,918
total length intr.int.s	1,173,284
fraction pos.s in intr.int.s	0.047
variability	0.104

no. runs big dataset	r	n/r
eBWT	547,991	45.881
dolEBWT	547,793	45.928
mdolBWT	555,687	45.276
concBWT	558,902	45.015
colexBWT	536,682	46.879
optBWT	533,712	47.140

Hamming d. / norm. Hamming d.	Hamming distance on a subset of 1,500 sequences			
	dolEBWT	mdolBWT	concBWT	colexBWT
dolEBWT	0	4,740	3,104	3,926
mdolBWT	0.00210	0	4,716	4,783
concBWT	0.00137	0.00209	0	4,208
colexBWT	0.00174	0.00212	0.00186	0

small dataset properties	
no. sequences	1,500
total length	2,260,229
average length	1,501
no. of interesting intervals	946
total length intr.int.s	72,933
fraction pos.s in intr.int.s	0.032
variability	0.104

edit d. / norm. edit d.	edit distance on a subset of 1,500 sequences				
	eBWT	dolEBWT	mdolBWT	concBWT	colexBWT
eBWT	0	18,328	22,194	21,021	21,987
dolEBWT	0.00811	0	4,410	2,761	3,858
mdolBWT	0.00982	0.00195	0	4,323	4,691
concBWT	0.00930	0.00122	0.00191	0	4,146
colexBWT	0.00973	0.00171	0.00208	0.00183	0

no. runs small dataset	r	n/r
eBWT	62,077	36.386
dolEBWT	62,031	36.437
mdolBWT	62,712	36.041
concBWT	62,800	35.991
colexBWT	61,235	36.911
optBWT	60,979	37.066

Table 7. Results for the 16S rRNA long dataset. First row left: absolute and normalized pairwise Hamming distance between separator-based BWT variants. First row right: summary of the dataset properties. Second row: number of runs and average runlength (n/r) of all BWT variants. Third row left: absolute and normalized pairwise Hamming distance between separator-based BWT variants on a subset of the input collection. Third row right: summary of the dataset properties of a subset of the input collection. Fourth row left: absolute and normalized pairwise edit distance between all BWT variants on a subset of the input collection. Fourth row right: number of runs and average runlength (n/r) of all BWT variants on a subset of the input collection.

Candida auris reads (50,000 long sequences)

Hamming d. / *norm. Hamming d.*	*Hamming distance on the big dataset*			
	dolEBWT	mdolBWT	concBWT	colexBWT
dolEBWT	0	306,071	306,431	305,665
mdolBWT	0.00246	0	305,649	305,713
concBWT	0.00247	0.00246	0	305,469
colexBWT	0.00246	0.00246	0.00246	0

dataset properties	
no. sequences	50,000
total length	124,200,880
average length	2,483
no. interesting intervals	39,076
total length intr.int.s	913,721
fraction pos.s in intr.int.s	0.007
variability	0.497

no. runs big dataset		
	r	n/r
eBWT	72,014,777	1.724
dolEBWT	71,972,783	1.726
mdolBWT	71,972,346	1.726
concBWT	71,973,221	1.726
colexBWT	71,725,274	1.732
optBWT	71,704,473	1.732

Hamming d. / *norm. Hamming d.*	*Hamming distance on a subset of 1,500 sequences*			
	dolEBWT	mdolBWT	concBWT	colexBWT
dolEBWT	0	6,333	6,393	6,260
mdolBWT	0.00169	0	6,411	6,354
concBWT	0.00170	0.00171	0	6,294
colexBWT	0.00167	0.00169	0.00168	0

small dataset properties	
no. sequences	1,500
total length	3,755,776
average length	2,503
no. of interesting intervals	1,189
total length intr.int.s	18,372
fraction pos.s in intr.int.s	0.005
variability	0.530

edit d. / *norm. edit d.*	*edit distance on a subset of 1,500 sequences*				
	eBWT	dolEBWT	mdolBWT	concBWT	colexBWT
eBWT	0	30,345	30,552	30,562	30,794
dolEBWT	0.00808	0	4,835	4,860	6,005
mdolBWT	0.00813	0.00129	0	4,824	6,105
concBWT	0.00814	0.00129	0.00128	0	6,035
colexBWT	0.00820	0.00160	0.00163	0.00161	0

no. runs small dataset		
	r	n/r
eBWT	2,635,300	1.425
dolEBWT	2,633,676	1.426
mdolBWT	2,633,652	1.426
concBWT	2,633,727	1.426
colexBWT	2,629,094	1.429
optBWT	2,628,470	$1,429$

Table 8. Results for the Candida auris reads dataset. First row left: absolute and normalized pairwise Hamming distance between separator-based BWT variants. First row right: summary of the dataset properties. Second row: number of runs and average runlength (n/r) of all BWT variants. Third row left: absolute and normalized pairwise Hamming distance between separator-based BWT variants on a subset of the input collection. Third row right: summary of the dataset properties of a subset of the input collection. Fourth row left: absolute and normalized pairwise edit distance between all BWT variants on a subset of the input collection. Fourth row right: number of runs and average runlength (n/r) of all BWT variants on a subset of the input collection.

SARS-CoV-2 genomes (2,000 long sequences)

Hamming d. / *norm. Hamming d.*	*Hamming distance on the big dataset*			
	dolEBWT	mdolBWT	concBWT	colexBWT
dolEBWT	0	7,958	7,900	7,263
mdolBWT	0.00013	0	7,958	7,957
concBWT	0.00013	0.00013	0	7,990
colexBWT	0.00012	0.00013	0.00013	0

dataset properties	
no. sequences	2,000
total length	59,612,692
average length	29,085
no. interesting intervals	1863
total length intr.int.s	80,486
fraction pos.s in intr.int.s	0.001
variability	0.148

no. runs big dataset		
	r	n/r
eBWT	117,628	506.773
dolEBWT	117,410	507.731
mdolBWT	118,870	501.495
concBWT	119,334	499.549
colexBWT	114,287	521.605
optBWT	113,930	523.240

Hamming d. / *norm. Hamming d.*	*Hamming distance on a subset of 50 sequences*			
	dolEBWT	mdolBWT	concBWT	colexBWT
dolEBWT	0	105	119	90
mdolBWT	0.00007	0	124	116
concBWT	0.00008	0.00008	0	118
colexBWT	0.00006	0.00008	0.00008	0

small dataset properties	
no. sequences	50
total length	1,490,184
average length	29,802
no. interesting intervals	43
total length intr.int.s	271
fraction pos.s in intr.int.s	$1.8 \cdot 10^{-4}$
variability	0.690

edit d. / *norm. edit d.*	*edit distance on a subset of 50 sequences*				
	eBWT	dolEBWT	mdolBWT	concBWT	colexBWT
eBWT	0	786	795	801	791
dolEBWT	0.00053	0	98	107	86
mdolBWT	0.00053	0.00007	0	105	112
concBWT	0.00054	0.00007	0.00007	0	114
colexBWT	0.00053	0.00006	0.00008	0.00008	0

no. runs small dataset		
	r	n/r
eBWT	25,258	58.997
dolEBWT	25,255	59.006
mdolBWT	25,274	58.961
concBWT	25,285	58.936
colexBWT	25,221	59.085
optBWT	25,210	59.111

Table 9. Results for the SARS-CoV-2 genomes dataset. First row left: absolute and normalized pairwise Hamming distance between separator-based BWT variants. First row right: summary of the dataset properties. Second row: number of runs and average runlength (n/r) of all BWT variants. Third row left: absolute and normalized pairwise Hamming distance between separator-based BWT variants on a subset of the input collection. Third row right: summary of the dataset properties of a subset of the input collection. Fourth row left: absolute and normalized pairwise edit distance between all BWT variants on a subset of the input collection. Fourth row right: number of runs and average runlength (n/r) of all BWT variants on a subset of the input collection.
